# Protein evolution in two co-occurring types of *Symbiodinium*: an exploration into the genetic basis of thermal tolerance in *Symbiodinium* clade D

**DOI:** 10.1186/1471-2148-12-217

**Published:** 2012-11-12

**Authors:** Jason T Ladner, Daniel J Barshis, Stephen R Palumbi

**Affiliations:** 1 Department of Biology, Stanford University, Hopkins Marine Station, 93950, Pacific Grove, CA USA

**Keywords:** Symbiodinium, Thermal tolerance, Coral, Symbiosis, Dinoflagellate, Transcriptome, Zooxanthellae, High-throughput sequencing, Symbiont shuffling

## Abstract

**Background:**

The symbiosis between reef-building corals and photosynthetic dinoflagellates (*Symbiodinium*) is an integral part of the coral reef ecosystem, as corals are dependent on *Symbiodinium* for the majority of their energy needs. However, this partnership is increasingly at risk due to changing climatic conditions. It is thought that functional diversity within *Symbiodinium* may allow some corals to rapidly adapt to different environments by changing the type of *Symbiodinium* with which they partner; however, very little is known about the molecular basis of the functional differences among symbiont groups. One group of *Symbiodinium* that is hypothesized to be important for the future of reefs is clade D, which, in general, seems to provide the coral holobiont (i.e., coral host and associated symbiont community) with elevated thermal tolerance. Using high-throughput sequencing data from field-collected corals we assembled, *de novo*, draft transcriptomes for *Symbiodinium* clades C and D. We then explore the functional basis of thermal tolerance in clade D by comparing rates of coding sequence evolution among the four clades of *Symbiodinium* most commonly found in reef-building corals (A-D).

**Results:**

We are able to highlight a number of genes and functional categories as candidates for involvement in the increased thermal tolerance of clade D. These include a fatty acid desaturase, molecular chaperones and proteins involved in photosynthesis and the thylakoid membrane. We also demonstrate that clades C and D co-occur within most of the sampled colonies of *Acropora hyacinthus*, suggesting widespread potential for this coral species to acclimatize to changing thermal conditions via ‘shuffling’ the proportions of these two clades from within their current symbiont communities.

**Conclusions:**

Transcriptome-wide analysis confirms that the four main *Symbiodinium* clades found within corals exhibit extensive evolutionary divergence (18.5-27.3% avg. pairwise nucleotide difference). Despite these evolutionary distinctions, many corals appear to host multiple clades simultaneously, which may allow for rapid acclimatization to changing environmental conditions. This study provides a first step toward understanding the molecular basis of functional differences between *Symbiodinium* clades by highlighting a number of genes with signatures consistent with positive selection along the thermally tolerant clade D lineage.

## Background

The symbiosis between stony corals (Scleractinia) and photosynthetic dinoflagellates in the genus *Symbiodinium* (zooxanthellae) allows for increased productivity in nutrient poor waters and forms the base of the coral reef ecosystem [1]. It is estimated that most corals receive up to 90% of their energy requirements from their symbionts [2]; meanwhile, the coral provides the dinoflagellates with shelter and inorganic nutrients necessary for photosynthesis [3]. This symbiosis is largely responsible for the success of stony corals over the last 200 Myr [4]; however, the future of this vital partnership is uncertain in the face of global climate change [5-7]. Many coral/zooxanthellae ‘holobionts’ (i.e.*,* cnidarian host and associated microbial community, *sensu*[8]) appear to be living at, or near, their upper thermal limits across a range of different regional thermal environments, with high temperature anomalies of just 1–2°C above mean local summer maximums known to cause disruption of photosynthesis, coral bleaching and even death [9-11]. Therefore, in the absence of significant acclimatization or adaptation, corals will likely face high levels of regional mortality over the next few decades [12-14].

One potential mechanism for corals to acclimatize to warming oceans is through a change in their symbiont partner (i.e., ‘shuffling’ or ‘switching’) [15,16]. Within *Symbiodinium* there are at least nine distinct phylogenetic clades (or subgenera) [17,18]. Four of these clades are commonly found in Scleractinian corals (clades A-D) and many coral species are known to associate with multiple symbiont clades, even within a single coral colony [19-21]. These four clades are estimated to have diverged between 30–65 Mya, and over this time they have accumulated important functional differences [10,18]. For example, clade A appears to have the unique ability to produce UV-protecting mycosporine-like amino acids [22], which may be partially responsible for its shallow distribution throughout the Caribbean [23] and its relatively enhanced ability to resist bleaching [24].

Clade D *Symbiodinium* are also of considerable interest because, in general, they appear to enhance the coral holobiont’s ability to deal with high temperatures. Evidence for the increased thermal tolerance of clade D comes from a variety of coral genera, geographic locations and data types. First, many studies have documented increased abundances of clade D *Symbiodinium,* across multiple genera of coral, in habitats characterized by unusually high temperatures (e.g., Palau: [25]; Thailand: [26]; American Samoa: [27,28]). Second, experimental work has demonstrated that, in multiple species, association with clade D *Symbiodinium* leads to reduced levels of bleaching and greater maintenance of photosynthetic efficiency during heat stress (*Pocillopora*: [29]; *Acropora*: [30,31]). Third, several studies examining symbiont communities pre- and post- natural bleaching events have demonstrated that corals hosting clade D survive in higher proportions than corals of the same species hosting clade C and that some corals change from clade C to clade D after bleaching (*Pocillopora*: [32]; *Acropora*: [33]). Although these patterns are not universal across all corals (e.g., [25,34]), the zooxanthellae in clade D [18] seem to possess functional differences, which allow clade D symbionts to provide greater thermal tolerance to the coral holobiont in many different geographic locations and host associations.

Despite the plethora of ecological and physiological data supporting increased thermal tolerance of clade D zooxanthellae, little is known about the particular adaptations that have led to this phenotype. One promising method for highlighting these functional differences is through the comparison of rates of protein sequence evolution (synonymous vs. non-synonymous substitutions) along closely related lineages (e.g., [35,36]). Here, we utilize high-throughput sequence data to construct partial transcriptome assemblies from natural populations of clades C and D extracted from field-collected samples of a single coral species (*Acropora hyacinthus*). We then examine rates of protein evolution along the C and D lineages with comparison to EST libraries from isolates of clades A and B [37]. These high-throughput sequences also provide us with a detailed view into the co-occurrence of clades C and D within colonies of *A. hyacinthus*, which has important implications regarding potential mechanisms for acclimatization via a change in symbiont communities.

We are able to highlight a number of genes and functional categories as candidates for involvement in the increased thermal tolerance of clade D. These include a fatty acid desaturase, molecular chaperones and proteins involved in photosynthesis and the thylakoid membrane. We also demonstrate that clades C and D co-occur within most colonies of *A. hyacinthus*, suggesting widespread potential for this coral species to acclimatize to changing thermal conditions via ‘shuffling’ the proportions of these two clades from within their current symbiont communities.

## Methods

### Sample collection

We obtained genetic material for *Symbiodinium* clades C and D directly from coral tissue collected from the field. Sixteen colonies of *A. hyacinthus* were sampled from the backreef pools on the south side of Ofu Island, American Samoa (14°11’S, 169°36’W). To increase the number of genes expressed (and therefore sequenced), two samples (~2 cm) were collected from each colony and transferred to nearby holding tanks, which were maintained at two distinct thermal regimes (one branch from each colony in each condition). One condition was maintained at ambient air temperature (26.8 – 34.5°C, mean = 29.2°C) while the temperature of the other condition was elevated by ~2.7°C (27 – 37.6°C, mean = 31.9°C). After 72 hours, all samples were preserved in a high-concentration trisodium citrate buffer.

### Extraction and sequencing

Total RNA was extracted from each sample using a modified TRIzol (GibcoBRL/Invitrogen, Carlsbad, CA, USA) protocol. Approximately 150-200 mg of coral tissue and skeleton was placed in 1 ml of TRIzol and homogenized for 2 min by vortexing with ~100 μl of 0.5 mm Zirconia/Silica Beads (BioSpec Products, Inc., Bartlesville, OK, USA). Resulting tissue/TRIzol slurry was removed by centrifugation and standard TRIzol extraction was performed according to manufacturer’s specifications with the replacement of 250 μl of 100% isopropanol with 250 μl of high salt buffer (0.8 M Na citrate, 1.2 M NaCl) during the final precipitation step. Resulting RNA pellet was resuspended in 12 μl of DEPC treated H_2_O.

Messenger-RNA (mRNA) was isolated from total RNA using the micro fast-track mRNA isolation kit (Invitrogen) and an overnight precipitation at −80°C. Between 40 ng and 1 μg of mRNA was used in Illumina library construction as in Beck *et al.*[38]. Briefly, mRNA was converted to double stranded cDNA in a PCR reaction containing random hexamer primers, Superscript III Reverse Transcriptase (Invitrogen) and supplied buffer. Reactions were cleaned with the MinElute Reaction Cleanup Kit (Qiagen, Valencia, CA USA). Double-stranded, paired-end oligonucleotide adapters were ligated onto the ds-cDNA using T4 DNA Ligase (Invitrogen) at 16°C for 4 hours. Resulting libraries were size selected for 200-300bp fragments using agarose gel electrophoresis and purified using the MinElute Gel Extraction Kit (Qiagen). The final library was generated by PCR amplification of the linker-ligated cDNA using P5 and P7 primers and Phusion PCR Master Mix (New England Biolabs, Ipswitch, MA, USA) using the following cycle program: initial denature at 98°C for 30 sec, 15 cycles of 98°C for 10 sec, 65°C for 30 sec, 72°C for 30 sec and a final extension at 72°C for 5 min.

In total, 31 libraries were constructed and sequenced using the Illumina Genome Analyzer II (Illumina; Additional file 1: Table S1). One of the elevated temperature samples (colony 3) was not sequenced due to poor RNA extraction. Seven of these libraries were sequenced by Illumina, Inc. (San Diego, CA) with a 76 base-pair (bp) paired-end sequencing length (152 bp per sequence total), four libraries were sequenced using single-end sequencing and a length of 36 bp in the lab of Dr. Arend Sidow (Stanford University), and the remaining 20 libraries were sequenced by Eureka Genomics (Hercules, CA). The latter 20 libraries were all done with single end sequencing, three were 72 bp reads the rest were 36 bp. An additional 36 bp paired-end lane was run for four of these libraries (colony 1, control and heated; colony 3, control; colony 9, heated) at the Stanford Functional Genomics Facility. These four additional lanes generated few reads due to concentration problems but are still incorporated in the following analyses.

Poor quality portions (Phred quality < 20) were trimmed from the ends of the raw sequences using the *FASTX-Toolkit*, and any reads < 20 bp were discarded (fastq_quality_trimmer -t 20 -l 20; http://hannonlab.cshl.edu/fastx_toolkit/). The *FASTX-Toolkit* was also used to remove any potential adapter sequences (fastx_clipper -l 20 -n).

### Symbiodinium community characterization

We estimated the proportion of each clade of *Symbiodinium* at the individual sample-level (i.e., mRNA library-level) by counting the abundance of clade specific reads at three loci that are known to be highly divergent between clades: internal transcribed spacer regions 1 and 2 (ITS1, ITS2) and chloroplast 23S rRNA (cp23S; Additional file 2: Table S2). Preliminary analyses, with reference sequences representing the full extent of *Symbiodinium* diversity, indicated that our coral samples contained only two types of *Symbiodinium,* one from clade C and the other from clade D (not shown). This is consistent with previous work done on these coral populations, which detected only a single genotype from each clade across 32 colonies of *A. hyacinthus* (using ITS1 and cp23S) [28]. Therefore, only these two types were considered in the final community characterizations (referred to in this manuscript as ‘clade C’ and ‘clade D’). Clade C and D ITS1 and cp23S reference sequences are from [28]. Clade-specific ITS2 sequences were mined from a preliminary *de novo* assembly of the data based on nucleotide similarity to the reported sequences from GenBank (NCBI). This resulted in two ITS2 sequences with best hits to type C3k (100% match) and type D2 (1bp different).

Each sequence library was mapped to these six clade-specific sequences using *BWA*[39]. Default settings were used except that we allowed for about 10% divergence between individual reads and the reference (−n 0.005), and to allow for an uneven distribution of mismatches within each read we increased the number of mismatches possible within the initial seeds (−k 5). These settings allow for sequence variability/sequencing error within populations while still preventing reads from aligning to the incorrect clade, and in practice we found these settings were sufficiently permissive to allow all reads from these loci to match (i.e., there was no substantial increase in the number of mapped reads with more permissive settings). For paired-end lanes, only the forward sequences were mapped. Duplicate reads were identified using *Picard* v1.43 (MarkDuplicates.jar; http://picard.sourceforge.net/), and clade proportions at each locus were calculated based on the number of well-mapped, non-duplicate reads (≥25 bp, mapping quality ≥30) to each of the clade-specific sequences, controlling for slight differences in sequence lengths between clades.

Each of these rRNA sequences is known to be multi-copy and therefore copy number differences between our *Symbiodinium* types, if present, could impact the number of rRNA sequences in our samples. However, by independently estimating proportions of the two clades from loci on two different genomes (nuclear and chloroplast), we have been able to demonstrate that any potential copy number differences have a minimal effect on our clade proportion estimates (see Results). In order to further validate these proportions, we also re-estimated the proportions of each clade by mapping all sequencing reads to the full transcriptomes that resulted from the *de novo* assembly (see below).

### De novo transcriptome assembly

Separate *de novo* assemblies were conducted for the two clades of *Symbiodinium*, using only the sequence libraries with ≥95% of a single *Symbiodinium* clade (averaged across the three clade-specific loci, see above). A small percentage of sequences from each non-focal clade are not expected to strongly bias the resulting assemblies because contigs were constructed using the consensus sequence at each base position. However, sequences originating from the non-focal clade may be incorporated into the transcriptome assembly if the two clades have highly divergent levels of expression (i.e., very low in the focal clade and very high in the non-focal clade). This situation is likely to be uncommon and most importantly will not result in significantly high dN/dS estimates.

The assemblies were conducted using CLC Genomics Workbench (v. 4, CLC Bio). Default setting were used except that the short read (<56 bases) penalties were adjusted to only allow for a maximum of five unaligned bases at the ends of reads, while still allowing for 2 mismatches or two indels (mismatch cost of 1, insertion and deletion costs of 2, limit of 5). Also the minimum length fraction of the long reads was decreased to 0.27 to help form contigs across low coverage regions. Our libraries contain a mixture of sequences from many members of the coral holobiont, including *Symbiodinium*, the cnidarian host and other associated microbes [8,40]. Therefore, we identified putative *Symbiodinum* contigs for each assembly based on sequence similarity (BLASTN ≥100bp, ≥75% identity or TBLASTX ≥50 amino acids, ≥85% identity) to ESTs from *Symbiodinium* sp. KB8 (clade A), sp. MF1.05b (clade B; http://medinalab.org/zoox/) and sp. C3 (clade C) [41], and *Polarella glacialis* (unpublished data) [42]*.* Potential coral contamination was then identified and removed based on sequence similarity (BLASTN ≥100bp, ≥75% identity) to a wide array of cnidarian cDNA databases: *A. hyacinthus* and *A. millepora* larval ESTs from the Matz Lab, UT-Austin [42] (http://www.bio.utexas.edu/research/matz_lab/matzlab/Data.html), *A. millepora* larval ESTs from the Miller lab, JCU [43], predicted transcripts from the *A. digitifera* genome [44] and predicted transcripts from the *Nematostella vectensis* genome [45]. Ribosomal RNA contamination was then removed based on significant nucleotide similarity (BLASTN, e-value ≤ 1×10^-8^) to the SILVA LSU and SSU rRNA databases (http://www.arb-silva.de/), and finally, MEGAN 4 [46] was used to remove any additional sequences likely to be contamination based on similarity to metazoans, fungi, bacteria or archaea. The MEGAN settings were altered slightly from the defaults: min. support = 1, min. score = 200, top percent = 20. These settings were chosen so that contigs would only be removed if they exhibited strong matches solely to groups that are distantly related to *Symbiodinium*. However, the settings were also conservative in that they only required a single match to a particular taxon for this taxon to be included in the analysis.

The remaining *de novo* contigs for clade C were then ‘meta-assembled’ based on sequence similarity to EST sequences from *Symbiodinium* sp. C3 [41] using custom python scripts. Our scripts join contigs based on nucleotide similarity to the same EST in a reference database. Our *de novo* contigs were joined if they had ‘good’ top blast hits (BLASTN, ≥100bp and ≥85% identity) to the same sp. C3 EST, if the reference sequence regions matched by the two contigs were overlapping or directly adjacent and if the contigs were ≥95% identical in any region of overlap. All scripts are available upon request.

### Contig annotation

*De novo* contigs were annotated to gene ontology (GO) categories based on translated amino acid similarity to the Swiss-Prot sequence database (BLASTX, e-value ≤1×10^-5^). Extraction of GO annotations from Uni-Prot flat files was automated using a custom perl script.

### Ortholog identification and sequence alignment

For the analyses of sequence divergence between clades, we also included publically available transcriptome assemblies from *Symbiodinium* sp. KB8 (clade A) and sp. MF1.05b (clade B) [37] (http://medinalab.org/zoox/). Protein sequences were identified and aligned identically for all four *Symbiodinium* clades. In general, we took caution at each step to avoid comparison of non-orthologous sequences. This inevitably means that extremely divergent genes and genes with closely related paralogs were excluded from our analyses.

Open reading frames (ORFs) and protein sequences were determined for each contig using *OrfPredictor*[47]. To minimize the number of erroneous protein predictions, we only utilized sequences if they fell into one of two categories: 1) significant similarity to a known protein sequence (BLASTX to NCBI’s nr, evalue ≤1×10^-5^) or 2) predicted protein sequence ≥200 amino acids long. The second category was included because exploratory analyses both utilizing and not utilizing BLASTX information have indicated that *OrfPredictor* is very accurate at identifying the correct reading frame for sequences with ≥200 amino acids, even without the use of BLASTX information (<5% error rate).

*InParanoid* v4.1 [48] was then used to identify orthologous sequences between clades based on protein-level similarity. *InParanoid* automates the identification of orthologs using a reciprocal BLAST based approach. In total, we conducted five pairwise comparisons among clades in order to construct 3- and 4-taxa ortholog groups: C-D, C-A, C-B, D-A and D-B. In each comparison, we also utilized protein sequences from three strains of *Toxoplasma gondii* (GT1, ME49 and VEG) [49] as an outgroup. If both sequences from a top ortholog pair in the C-D analysis shared the same top ortholog match in clade B, these three sequences were combined into a 3-taxa ortholog group (BCD). Similarly, if the same C and D sequences also shared the same top A ortholog, a 4-taxa ortholog group was formed (ABCD). Ortholog pairs from C-D with no shared clade B ortholog were treated as 2-taxa ortholog groups (CD). Orthologous protein sequences were aligned using *MUSCLE*[50] and *PAL2NAL*[51] was used to construct nucleotide alignments. PAL2NAL was also used to remove gaps and in frame stop codons. Levels of nucleotide sequence divergence between clades were calculated for each ortholog group in a pairwise manner using custom python scripts; positions with ambiguous bases were not included in these calculations.

### Rates of protein evolution

*PAML* v4 [52] was used to explore rates of synonymous and non-synonymous nucleotide substitutions in a phylogenetic context (d_N_/d_S_ or ω). The d_N_/d_S_ ratio is one of the most fundamental and powerful tests for identifying genes that have been affected by positive selection because it highlights loci with higher than expected rates of non-synonymous substitution. However, a high d_N_/d_S_ ratio can be also achieved by lower than expected levels of synonymous substitution. Therefore, d_N_/d_S_ results, in general, should be interpreted with an appropriate level of caution.

For 2-taxa ortholog groups, the *codeml* package was used to estimate levels of d_N_/d_S_. With only two taxa, it is not possible to polarize rates of evolution along specific branches; however, these rates are able to highlight genes with generally high levels of amino acid substitutions, which may have been affected by positive selection in one or both clades. We only report results of analyses with a total tree length ≤4 (# substitutions per codon).

**Figure 1:**
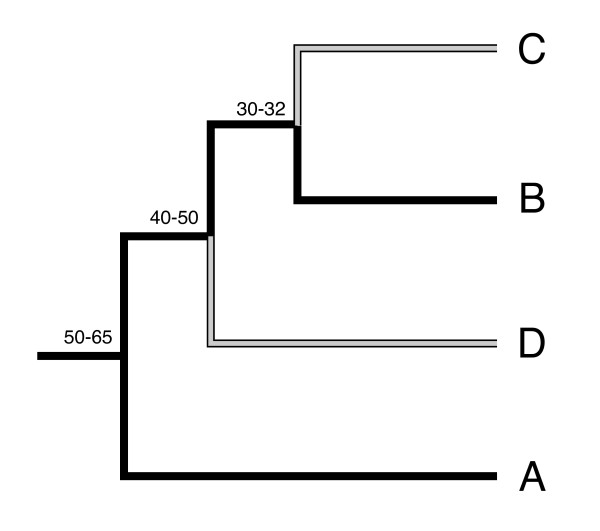
**Phylogenetic tree of *Symbiodinium *clades A-D used in *PAML *analyses investigating rates of amino acid sequence evolution.** Grey branches indicate the focal lineages leading to *Symbiodinium* clades **C** and **D**. Numbers indicate estimated divergence times (in millions of years) based on [10,18].

For the 3- and 4-taxa ortholog groups, branch-models in the *codeml* package were used to separately test for significantly different rates of d_N_/d_S_ along the clade C and clade D lineages. Figure 1 illustrates the phylogenetic tree used in these analyses (based on [53]). Specifically, for each group of orthologs we first ran an analysis with a single ω (d_N_/d_S_) across the entire tree (model=0). This first analysis served as our null model and was also used to estimate the degree of divergence between orthologs (i.e., total tree length). Simulation studies have demonstrated that *PAML* is able to remain informative even with large amounts of sequence divergence, especially when the phylogenetic tree contains many branches [54,55]. Regardless, we chose to be somewhat conservative by setting a maximum tree length cutoff (# substitutions per codon) of 2 times the number of taxa in the tree. This resulted in a maximum tree length of 8 for 4-taxa analyses and 6 for 3-taxa analyses. If a 4-taxa ortholog group had a tree that did not meet this threshold, it was then run as a 3-taxa analysis. Similarly, if a 3-taxa group had a tree that was too long, it was run as a 2-taxa analysis. In addition to focusing the analysis on the most informative subset of the data, these cutoffs also helped to screen for poor nucleotide alignments.

For each ortholog group that passed our tree length cutoff, we then ran two analyses to look for contigs evolving at different rates along our focal lineages: 1) one rate along the clade C lineage (ω_C_) and a second rate in the rest of the tree (ω_BG_) and 2) one rate along the clade D lineage (ω_D_) and a second rate in the rest of the tree (ω_BG_). Likelihood ratio tests were used to examine whether each of these two-rate models fit the data significantly better than the null, single-rate model using a χ^2^ distribution with one degree of freedom. These analyses resulted in four subset lists of ortholog groups, which serve as candidates for lineage-specific natural selection: 1) significantly (p<0.05) higher d_N_/d_S_ along the clade C lineage, 2) significantly lower d_N_/d_S_ along the clade C lineage, 3) significantly higher d_N_/d_S_ along the clade D lineage and 4) significantly lower d_N_/d_S_ along the clade D lineage. GOEAST [56] was used to look for enrichment of specific gene ontology categories within each of these ortholog lists. Default parameters were used in these enrichment analyses.

## Results

### Transcriptome sequences

The number of sequences was highly variable among Illumina runs. On average, we obtained ~10.8 million reads per single-end lane and ~24.4 million reads per paired-end lane after quality trimming and adapter clipping. This equates to 2–37 million reads and 0.067-2 billion base pairs per sequence library (i.e., coral sample; Additional file 1: Table S1).

### Symbiodinium community compositions and de novo transcriptome assembly

A total of 20–704 non-duplicate reads (mean=348) mapped to our three clade-specific markers per library, and the estimated *Symbiodinium* clade proportions were highly consistent across the three markers (avg. standard deviation across markers =2.8%; Table 1 and additional file 3: table S3). Furthermore, these 3-marker based estimates of clade proportions are highly consistent with proportions that were later estimated by mapping reads from each library to the full *de novo* transcriptomes of the two clades (R^2^=0.985; Additional file 4: Figure S1). Therefore, we believe these to represent accurate descriptions of the compositions of our symbiont communities.

**Table 1:** ***Symbiodinium *** **community characterizations for each coral sample, including estimated proportion of clade D at three different loci, the average and standard deviation across the three proportions and the total number of sequence reads used in these estimations**

**Colony**	**Treatment**	**Proportion Clade D**				**Standard deviation**	**Total # reads**
		**ITS1**	**ITS2**	**cp 23S**	**Average**		
1*	control	1.000	1.000	1.000	1.000	0.000	226
1*	heated	0.994	1.000	1.000	0.998	0.004	268
2*	control	0.973	1.000	1.000	0.991	0.015	206
2*	heated	1.000	1.000	1.000	1.000	0.000	169
3*	control	0.990	1.000	0.984	0.991	0.008	389
6*	control	0.974	1.000	0.938	0.971	0.031	236
6*	heated	1.000	0.984	0.879	0.954	0.065	123
9*	control	0.974	1.000	1.000	0.991	0.015	126
9*	heated	0.990	1.000	0.981	0.990	0.009	348
31^	control	0.013	0.000	0.006	0.006	0.006	645
31^	heated	0.006	0.000	0.009	0.005	0.005	519
40^	control	0.000	0.000	0.000	0.000	0.000	219
40^	heated	0.011	0.024	0.000	0.012	0.012	202
44*	control	0.993	0.977	0.986	0.986	0.008	507
44^	heated	0.005	0.000	0.005	0.003	0.003	632
45	control	0.091	0.274	0.336	0.234	0.128	168
45^	heated	0.037	0.074	0.000	0.037	0.037	69
55^	control	0.039	0.013	0.064	0.038	0.026	704
55^	heated	0.031	0.000	0.036	0.022	0.020	636
61	control	0.439	0.387	0.587	0.471	0.104	521
61	heated	0.785	0.782	0.789	0.785	0.004	621
65^	control	0.000	0.012	0.038	0.017	0.020	240
65^	heated	0.000	0.000	0.000	0.000	0.000	583
68	control	0.361	0.108	0.099	0.189	0.149	77
68^	heated	0.000	0.000	0.000	0.000	0.000	290
70^	control	0.011	0.022	0.057	0.030	0.024	391
70^	heated	0.005	0.039	0.035	0.026	0.018	380
71*	control	0.983	1.000	0.952	0.978	0.024	563
71*	heated	0.980	0.992	0.943	0.971	0.025	599
75	control	1.000	1.000	0.829	0.943	0.098	101
75	heated	1.000	1.000	1.000	1.000	0.000	20

For read count breakdowns for each individual locus see Additional file 3: Table S3. The symbols next to colony numbers indicate samples that were used in the *de novo* transcriptome assemblies: ^ for clade C *Symbiodinium*, * for clade D. ITS1 & ITS2=internal transcribed spacer regions 1 and 2, cp 23S=chloroplast 23S rRNA.

Thirteen sequence libraries had an average estimated proportion of clade C ≥95% (mean=98.5%). These 13 libraries consist of 5 control tank samples and 8 heated tank samples and contain ~233 million reads; all 13 were used in the *de novo* assembly of the clade C transcriptome. An additional 13 libraries had average estimated proportions of clade D ≥95% (mean=98.5%). However, one of these libraries (colony 75, heated) had only 20 reads align in total to all six clade-specific sequences. Due to low confidence in clade assignment from so few sequences, this library was not included in the clade D transcriptome assembly. The remaining 12 libraries consist of 7 control tank branches and 5 heated tank branches and contain ~239 million reads. After BLAST-based filtering and joining (Additional file 5: Table S4), the clade C assembly consists of 26,986 contigs (mean length=464, N50=534) and the clade D assembly contains 23,777 contigs (mean length=698, N50=920). A total of 9184 clade C contigs (34.0%) had good BLASTX hits to 6626 different proteins in Swiss-Prot, and 10,629 of the clade D contigs (44.7%) had good hits to 7374 proteins.

### Ortholog identification and clade divergence

**Figure 2:**
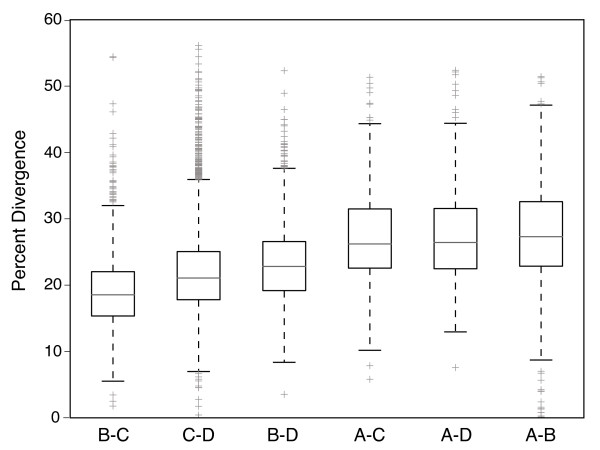
**Pairwise nucleotide sequence divergences between *Symbiodinium *clades A-D at the 611–3557 protein coding loci used in analyses investigating rates of amino acid sequence evolution.** Divergences represent raw sequence differences without correction for multiple mutations at a single base.

*InParanoid* analyses identified 758 4-taxa orthologs (ABCD), an additional 645 3-taxa orthologs (BCD) and 3437 ortholog groups with sequences from only clades C and D. After filtering based on total tree length (# substitutions per codon), 611 ortholog groups were analyzed with all 4 taxa represented (average tree length = 4.1) and 528 were analyzed with 3 taxa (average tree length = 2.5). The remaining 3701 ortholog groups were run as pairwise comparisons between clades C and D; however, only 2418 of these were under our maximum tree length threshold (average tree length = 1.7). Nucleotide alignments averaged 635 nucleotides in length for the 4-taxa orthologs (min=147, max=1857), 525 nucleotides for the 3-taxa orthologs (min=108, max=1419) and 573 nucleotides for the 2-taxa orthologs (min=81, max=4377). Levels of nucleotide divergence between clades were highly variable across orthologs, but, in general, levels of divergence were high with median divergences among clades ranging between 18.5-27.3% (Figure 2). Based on divergence time estimates from Pochon et al. [18], these divergences equate to an overall substitution rate of ~3.1-3.4×10^-9^ per site per year

### Rates of protein evolution

**Figure 3:**
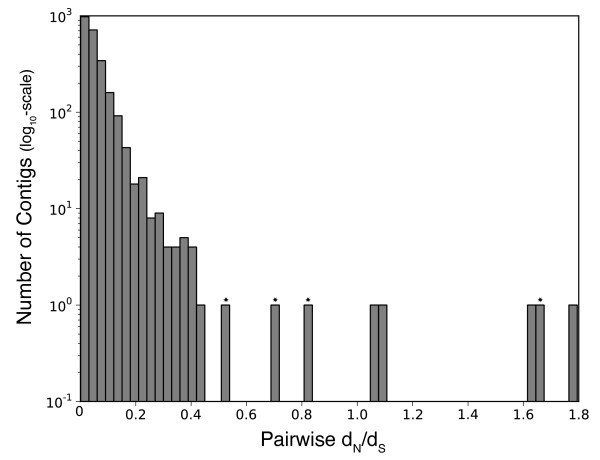
**Histogram depicting the distribution of pairwise d**_**N**_**/d**_**S **_**estimates comparing clades C and D across 2418 protein coding sequences.** Stars indicate qualitatively outlier d_N_/d_S_ values for sequences with significant sequence similarity to annotated proteins in Swiss-Prot. Table 2 contains information on these annotations.

From the 2418 pairwise analyses, d_N_/d_S_ could not be estimated in three instances because d_S_=0 (d_N_ was also 0 in two cases). For the rest, d_N_/d_S_ was highly skewed toward low values (mean=0.057; Figure 3); however, eight ortholog pairs were qualitatively distinguishable from the core of the distribution, and five of these had d_N_/d_S_ >1 (Table 2). Four of these qualitative outliers had Swiss-Prot annotations in at least one of the *Symbiodinium* clades. These included an avidin-related protein, a RING finger protein, a magnesium-chelatase subunit and a protein with disulfide oxidoreductase activity (Table 2).

**Table 2:** **Qualitative outliers from the pairwise estimates of d**_**N**_**/d**_**S **_**between clades C and D *Symbiodinium***

**d**_**N**_**/d**_**S**_	**Swiss-Prot annotation**		**Contig names**	
	**Clade C**	**Clade D**	**Clade C**	**Clade D**
1.79	--	--	c_sym_54689	d_sym_6384
1.65	Avidin-related protein 4/5	Avidin-related protein 7	c_sym_66085	d_sym_105906
1.64	--	--	c_sym_32989	d_sym_105290
1.08	--	--	c_sym_32766	d_sym_105256
1.05	--	--	c_sym_98918	d_sym_110077
0.83	--	Magnesium-chelatase 30 kDa subunit	c_sym_11466	d_sym_36635
0.71	RING-H2 finger protein ATL1	RING finger protein 32	c_sym_38569	d_sym_8618
0.54	Thioredoxin domain-containing protein	Protein disulfide-isomerase A2	c_sym_69335	d_sym_71587

Dashes indicate no hit to Swiss-Prot with e-value<1×10^-5^.

Of the 1139 multi-branch *codeml* analyses (ABCD and BCD), 207 orthologs (18%) were found to have significantly different rates of d_N_/d_S_ along the clade D lineage (p<0.05; Additional file 6: Table S5-Additional file 7: Table S6); 74 (6.4%) had higher than average rates, while 133 (11.6%) exhibited rates that were lower than average. Of these, 48 loci (23%) remained significant after Benjamini-Hochberg false discovery rate (FDR) correction (α=0.05) [57] and 136 (65.7%) remained significant after Sequential Goodness of Fit (SGoF) correction (α=0.05) [58] (Additional file 6: Table S5-Additional file 7: Table S6). Similarly, 154 (13.4%) of the same orthologs had significantly different rates along the C lineage (p<0.05), 88 (7.7%) were higher than average and 66 (5.7%) were lower (Additional file 8: Table S7-Additional file 9: Table S8). Of these, 11 loci (7.1%) remained significant after Benjamini-Hochberg FDR correction (α=0.05) [57] and 83 (53.9%) remained significant after SGoF correction (α=0.05) [58] (Additional file 8: Table S7-Additional file 9: Table S8).

**Figure 4:**
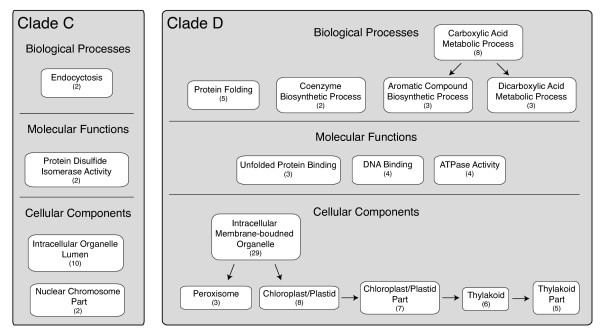
**Gene ontology (GO) categories exhibiting enrichment (p<0.05, uncorrected) for orthologs with significantly elevated d**_**N**_**/d**_**S **_**along the clade C and D lineages, respectively.** If multiple hierarchical categories were all enriched and included the exact same subset of orthologs, only the most specific of these categories is shown. Numbers indicate the number of orthologs with significantly elevated d_N_/d_S_ in each category. The arrows connect categories that contain a subset of the orthologs found within other enriched categories. These connections do not necessarily represent GO hierarchical relationships.

Our power to detect significantly different rates of d_N_/d_S_ may be somewhat eroded due to the high levels of nucleotide divergence among clades (Figure 2); therefore, for the functional enrichment analyses we chose to utilize all orthologs that were significant prior to multiple test correction. This allows us to generally explore the categories of genes exhibiting signatures of elevated and depressed d_N_/d_S_, even if the signatures of selective events have been somewhat masked by the accumulation of large amounts of synonymous divergence. Multiple gene ontology categories were significantly (p<0.05) enriched in each of our four focal gene lists prior to correction for multiple tests (Additional file 10: Table S9; for summary of high d_N_/d_S_ lists see Figure 4). None of these categories remain significantly enriched after correcting for multiple tests. However, these nearly significant enrichment categories still provide a good view of the types of genes exhibiting nucleotide substitutions characteristic of differential selection pressures on the two focal lineages. Therefore, we explore these enriched categories below.

## Discussion

### Genetic divergence between clades

Phylogenetic studies looking at a handful of highly-polymorphic, non-protein coding genetic markers have demonstrated strong evolutionary divergence between *Symbiodinium* clades, and based on these sequences, divergence times between clades A-D have been estimated at ~30-65 million years (Figure 1) [10,18,53]. However, little is known about genome-wide levels of divergence, especially at protein-coding genes, which typically experience higher levels of mutational constraint (though see [59]). Our results illustrate substantial genetic divergence throughout most coding genes between all four of the clades most commonly found within Scleractinian corals (A-D), with raw median nucleotide divergences ranging from 18.5-27.3% and Kimura 2-parameter corrected distances [60] of 21.6-34.3% (Figure 2). Although sequencing and alignment errors may be adding to these high levels of divergence, there is also reason to believe that these divergences may actually be underestimates because we only calculated sequence divergence at loci that were similar enough, at the amino acid level, to be identified as orthologs and similar enough, at the nucleotide level, to fall within our maximum tree length cutoffs. Additionally, these estimates are based only on single nucleotide polymorphisms (SNPs); they do not include insertion and deletion differences.

It has been a long debate in the *Symbiodinium* research community as to what level of divergence these clade designations represent (e.g., species, genus, family); however, accurate characterization of genome wide divergence has been limited by the lack of genomic resources available for these taxa. The primates represent one well-studied system for which several similar estimates of genomic divergence are available. George et al. [61] report exome-wide divergences between *Homo sapiens* and seven non-human primates spanning three families and seven genera. The average inter-genera divergence is 2.35% and the average inter-family divergence is 2.91%. The lowest level of differentiation estimated in our study, between clades B and C (putatively belonging to the same genus), is more than four times higher than the highest level of differentiation reported by George et al. (4.19% between human and tamarin) [61]. Although the use of taxonomic divisions is not standardized among groups, the high levels of genomic divergence seen among these four *Symbiodinium* clades certainly suggests that they should be recognized at some higher level of taxonomic division, such as family or order. This is consistent with early phylogenetic work based on rDNA sequences, which demonstrated that within a single locus, genetic diversity within *Symbiodinium* was equivalent to order-level differences seen in other dinoflagellate groups [62].

The observed relative levels of genomic differentiation are consistent with previously estimated phylogenetic relationships [18,53], with clades B and C exhibiting the lowest level of divergence, clade D showing intermediate levels of divergence to B and C, and clade A showing high levels of divergence to all other clades. Assuming a 10–30 day generation time, *in hospite*[63,64]*,* the resulting estimate for *Symbiodinium* substitution rates (~3.1-3.4×10^-9^/site/year) equates to ~3.8-12.5×10^-8^ substitutions per site per generation. This is fairly consistent with mutation rate estimates for *Caenorhabditis elegans* and *Drosophila*: 2.1×10^-8^ and 0.8×10^-8^ per site per generation, respectively (reviewed in [65]).

### Genes with elevated d_N_/d_S_ along the clade D lineage

One of the best-described biochemical differences between relatively heat tolerant and sensitive types of *Symbiodinium* is related to the saturation state of the thylakoid lipid membranes of the chloroplast. Tchernov et al. [10] demonstrated that several different relatively heat tolerant strains of zooxanthellae (isolates from clades A, B and E) had thylakoid membranes with substantially lower proportions of unsaturated polyunsaturated fatty acids making them more thermally stable and less susceptible to attack by reactive oxygen molecules. No clade D symbionts were included in the analysis of Tchernov et al. [10], but given the generality of their results across relatively thermally tolerant subtypes in clades A, B and E, saturation state of the thylakoid membranes is likely to be important in clade D as well. Our analysis revealed one desaturase involved in unsaturated fatty acid synthesis with significantly elevated d_N_/d_S_ along the clade D lineage (4-taxa ortholog 515, ω_D_=0.094, ω_BG_=0.041; Additional file 6: Table S5). This ortholog is very similar to the palmitoyl-monogalactosyldiacylglycerol delta-7 desaturase (FAD5) in *Arabidopsis thaliana* (BLASTX evalue=3×10^-104^), which is involved in early stages of desaturation of fatty acids used in the synthesis of thylakoid membranes [66]. A nonsense mutation in this gene in *A. thaliana* resulted in a change in the composition of the lipid thylakoid membrane, a reduction in chlorophyll content and delayed recovery of photosystem II after photoinhibition by high light stress [66,67]. Therefore, this gene is a strong candidate for involvement in the described functional differences between our clade C and D type *Symbiodinium*[31]. No genes involved in fatty acid synthesis exhibited elevated d_N_/d_S_ along the clade C lineage.

In addition to this fatty acid desaturase, seven additional genes related to the chloroplast exhibited elevated d_N_/d_S_ along the clade D lineage (4 significant after SGoF; Figure 4, Additional file 10: Table S9), including five that compose parts of the thylakoid membrane. Based on protein matches in Swiss-Prot, these orthologs include two subunits of the photosystem I reaction center (subunit II = 4-taxa ortholog 707, ω_D_=0.118, ω_BG_=0.021; subunit IV = 4-taxa ortholog 202, ω_D_=0.153, ω_BG_=0.032; Additional file 6: Table S5), part of the light-harvesting complex of photosystem II (Fucoxanthin-chlorophyll a-c binding protein F = 4-taxa ortholog 352, ω_D_=infinite, ω_BG_=0.044; Additional file 6: Table S5) and a membrane protein involved in the insertion of proteins into the inner membrane of the thylakoid (Inner membrane ALBINO3-like protein 2 = 3-taxa ortholog 58, ω_D_=0.078, ω_BG_=0.04; Additional file 7: Table S6). In contrast, we found only one gene involved in photosynthesis with elevated d_N_/d_S_ along the clade C lineage, peridinin-chlorophyll a-binding protein (3-taxa ortholog 433, ω_D_=infinite, ω_BG_=0.046; Additional file 9: Table S8). Therefore, proteins related to the thylakoid and photosynthesis appear to be additional candidates for functional differences between clade C and D *Symbiodinium*.

Several additional functional categories of interest are marginally enriched within the orthologs exhibiting elevated d_N_/d_S_ along the clade D lineage, including the biological process category ‘protein folding’ (5 orthologs; 4 significant after SGoF) and the related molecular function category ‘unfolded protein binding’ (i.e., chaperones, 3 orthologs), which include matches to heat-shock protein 90 (3-taxa ortholog 1308, ω_D_=infinite, ω_BG_=0.0001; Additional file 7: Table S6), prefoldin subunit 3 (4-taxa ortholog 424, ω_D_=0.099, ω_BG_=0.028; Additional file 6: Table S5) and chaperone protein DnaJ (4-taxa ortholog 365, ω_D_=infinite, ω_BG_=0.018; Additional file 6: Table S5). Chaperones are known to have a diverse set of non-stress related cellular roles, primarily involved in preventing inappropriate interactions among proteins in non-native conformations [68]. However, it is also well documented that chaperone proteins are often upregulated in response to a multitude of stressors, including high temperatures, which can cause protein denaturation [68]. To our knowledge there has not yet been any large-scale gene expression study dedicated to *Symbiodinium* (reviewed in [69]). Therefore, it is unclear whether these chaperones are also upregulated in response to heat stress in zooxanthellae. However, Leggat et al. [41] identified heat-shock protein 90 as the fourth most abundant transcript in an EST library constructed for *Symbiodinium* sp. C3 extracted from *Acropora aspera*. In contrast to these results, no chaperones or protein binding genes were identified with elevated d_N_/d_S_ along the clade C lineage. Higher rates of amino acid evolution of these proteins in clade D may be related to the higher thermal tolerance of clade D *Symbiodinium*.

It is worth noting that a number of the orthologs exhibiting significantly high d_N_/d_S_ along the clade D lineage also show significantly low d_N_/d_S_ along the clade C lineage (Additional file 11: Table S10). This result emphasizes the different patterns of evolution along these two lineages, but it also a good reminder that the analyses of d_N_/d_S_ along our two focal branches are not independent. Therefore, from the current analysis it is not possible to determine whether the significant results are due to stronger than average positive selection along the D lineage, stronger than average purifying selection along the C lineage or some combination of the two. It is, of course, also possible that rates of change along additional braches of the tree could be influencing the patterns described. Furthermore, temperature tolerance is just one phenotypic difference that exists between clades C and D; therefore, genes involved in temperature tolerance are expected to represent only a subset of the loci highlighted in these analyses. However, the main purpose of this study is to identify candidate genes for the functional differences between clades C and D. Further work is necessary to explore each of these candidates in detail and to elucidate what, if any, role they may have played in the thermal adaptation of clade D *Symbiodinium*.

Additional genes that have been under positive selection along the C and D lineages may have been missed due to high levels of synonymous sequence divergence among clades, which can mask signatures of selection. High levels of sequence divergence appear to be inevitable when comparing *Symbiodinium* clades in Scleractinian corals (Figure 2); however, studies have begun to document high levels of functional divergence even within *Symbiodinium* clades and sub-clade types (e.g., [10,28,34,70]). For example, type C1 has been shown to be even more thermally tolerant than some clade D symbionts in association with particular coral species [34], and similarly heat-tolerant *Symbiodinium* types have also been described in clades A and B [10]. Subclade-level genetic comparisons within clade D will be critical for pinpointing the exact evolutionary lineage(s), within clade D, on which selection for increased heat-tolerance has occurred. This type of analysis is also likely to highlight additional genes involved in thermal tolerance of the clade D type evaluated in this study. Similar subclade-level studies in the other major coral-associated *Symbiodinium* clades will also be important to see if similar mechanisms are involved in heat-tolerance among the different clades. Increased heat-tolerance may have evolved multiple independent times within *Symbiodinium*, and therefore, heat-tolerant types within the different clades may have evolved different mechanisms for coping with increased temperatures.

### Mixed assemblages of symbiodinium

The majority of genotyping methods for *Symbiodinium* have a detection threshold for background types of >5-10% of the total symbiont population [20,21,71]. Quantitative real-time PCR techniques have demonstrated that because of this limitation, traditional methods have greatly underestimated the number of corals hosting multiple clades of *Symbiodinium*[20]. Our characterizations of symbiont communities using high-throughput sequencing data provide further evidence for the prevalence of background strains of *Symbiodinium* at low frequencies. All 16 colonies of *Acropora hyacinthus* sampled in this study exhibit some proportion of both clades C and D when results are pooled across the two samples from each colony (background frequencies: 0.1-49.4%), despite the fact that PCR-based cp23S genotyping detected only a single clade in each of these colonies. The ubiquitous presence of both clades C and D within *A. hyacinthus* colonies in Ofu illustrates the strong potential within this population for symbiont ‘shuffling’ (i.e., changing the proportions of different symbionts already coexisting within a coral colony) to play a role in acclimatization of individual colonies in response to environmental fluctuations.

It is possible that this level of co-occurrence of clades C and D is unusual for corals. American Samoa has been shown to have a relatively high incidence of both clades C and D [27]. By contrast, type D is less abundant in many other Pacific localities [27]. Co-association with both clades C and D may be less prevalent in situations where one clade is particularly rare. However, Mieog et al. [20] demonstrated similarly high levels of co-occurrence of clades C and D in four species of coral across 11 geographic locations on the Great Barrier Reef. Therefore, high-levels of co-occurrence are clearly not unique to American Samoa.

A surprise in our work is the suggestion that different parts of the same colony house different proportions of symbiont clades C and D (Table 1). Symbiont community compositions are known to systematically vary within colonies of some coral species based on irradiance gradients (e.g., tops vs. sides of colonies in *Montastrea* spp. in the Caribbean; [24]). However, *A. hyacinthus* in Ofu, American Samoa occur as flat plates ~20-60 cm across with little obvious depth or light heterogeneity from branch to branch. To date, we can detect no clear environmental gradient within plates that would explain large differences in *Symbiodinium* community compositions across a colony.

## Conclusion

Transcriptome-wide analysis confirms the presence of deep evolutionary divisions among the four most common *Symbiodinium* clades associated with reef-building corals. Nevertheless, phylogenetic tree-based comparisons of relative rates of nucleotide substitutions highlight a number of genes and functional categories that are candidates for the functional differences that have been attributed to clade D *Symbiodinium*. Top candidates include a fatty acid desaturase, three molecular chaperones and several proteins involved in photosynthesis and the thylakoid membrane. These data provide the first genomic-scale exploration into the adaptive thermotolerance of clade D *Symbiodinium*. The use of high-throughput sequencing data from field-collected corals also allowed for a detailed examination into the composition of naturally occurring *Symbiodinium* communities. Our results provide additional support for the prevalence of multiple *Symbiodinium* clades within individual coral colonies, and therefore the capacity of individual colonies to adjust to changing conditions by ‘shuffling’ the proportions of their resident endosymbionts.

## Database submission

The *de novo* assembled contigs used in this study have been deposited in the NCBI Transcriptome Shotgun Assembly database under the accession numbers GAFO00000000 (Clade C) and GAFP00000000 (Clade D). The versions described in this paper are the first versions, GAFO01000000 and GAFP01000000, respectively. They are also available for download on the Palumbi lab website: http://palumbi.stanford.edu/data/.

## Competing interests

The authors declare that there are no competing interests.

## Authors’ contributions

JTL conceived and designed the project, conducted the data analysis and drafted the manuscript. DJB helped conceive the project, processed many of the samples for sequencing and helped write the manuscript. SRP helped conceive and design the project and write the manuscript. All authors read and approved the final manuscript.

## Supplementary Material

Additional file 1: Table S1 Total number of sequence reads and average read length for each coral branch after quality trimming and adapter clipping. Click here for file

Additional file 2: Table S2 Clade specific sequences used to quantitatively characterize *Symbiodinium* communities using high-throughput transcriptome sequence data. Click here for file

Additional file 3: Table S3 *Symbiodinium* community characterizations with read counts broken down by individual loci. Click here for file

Additional file 4: Figure S1 Correlation between estimates of *Symbiodinium* community composition. Click here for file

Additional file 5: Table S4 Read/contig counts at each step along the process of obtaining high-quality *Symbiodinium de novo* assemblies. Click here for file

Additional file 6: Table S5 4-taxa (ABCD) analyses testing d_N_/d_S_ along the clade D lineage. Click here for file

Additional file 7: Table S6 3-taxa (BCD) analyses testing d_N_/d_S_ along the clade D lineage. Click here for file

Additional file 8: Table S7 4-taxa (ABCD) analyses testing d_N_/d_S_ along the clade C lineage. Click here for file

Additional file 9: Table S8 3-taxa (BCD) analyses testing d_N_/d_S_ along the clade C lineage. Click here for file

Additional file 10: Table S9 Results of the Gene Ontology enrichment analyses of four different subsets of orthologs. Click here for file

Additional file 11: Table S10 Loci exhibiting significantly elevated and/or depressed d_N_/d_S_ along both the clade C and clade D lineages. Click here for file
