# Supplementary material for: Protein evolution in two co-occurring types of Symbiodinium: an exploration into the genetic basis of thermal tolerance in Symbiodinium clade D
Source: BMC Evol Biol. 2012 Nov 12;12:217. doi: 10.1186/1471-2148-12-217 (PMC3740780; doi:10.1186/1471-2148-12-217)
Supplement: Additional file 4: Figure S1 — Correlation between estimates of Symbiodinium community composition. [file 1471-2148-12-217-S4.pdf]

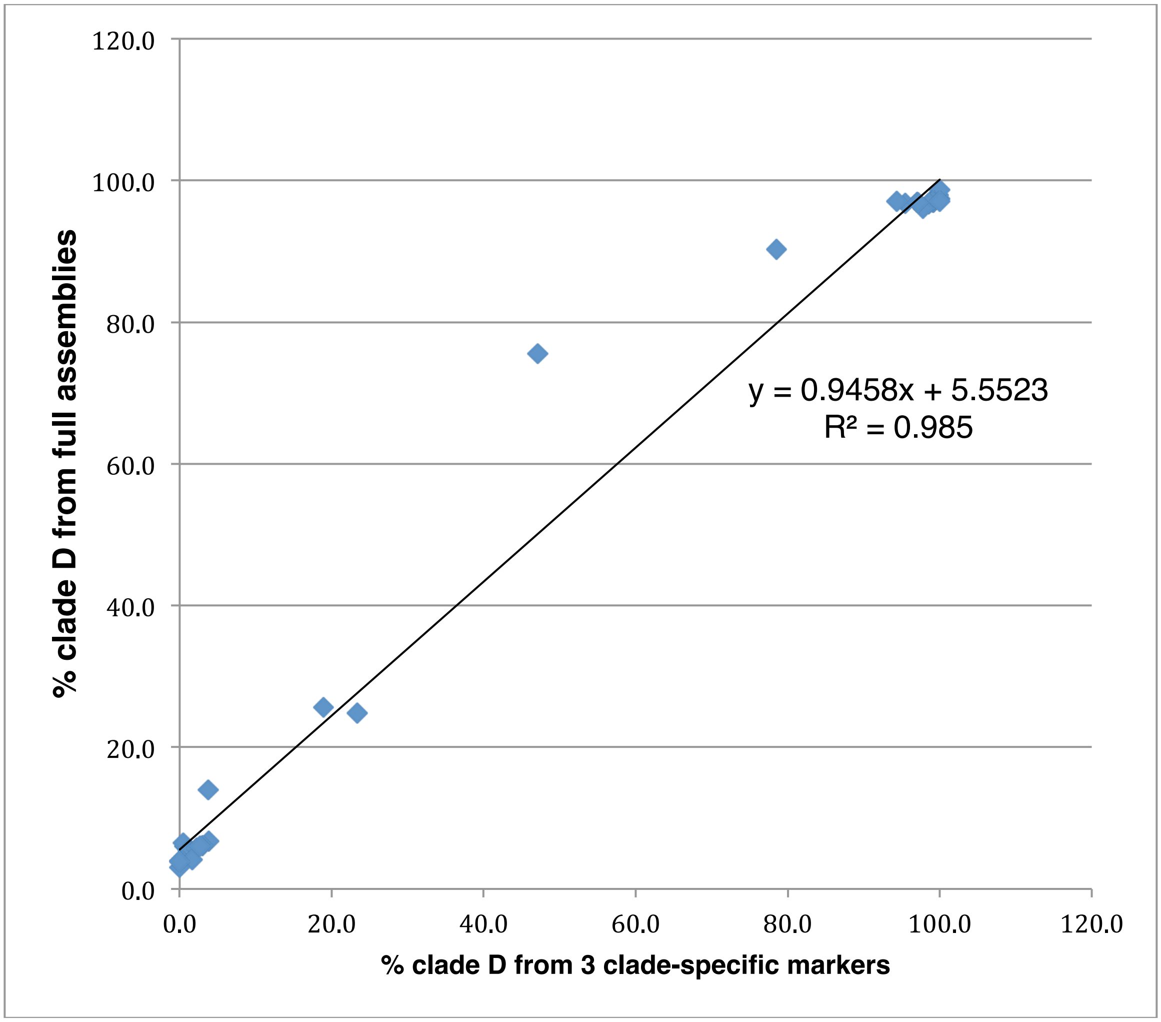

Figure S1. Correlation between two different estimates of the percentage of clade D *Symbiodinium* in each coral sample. On the x-axis is an estimate based on the number of reads aligning to three clade-specific markers (chloroplast 23S ribosomal RNA and internal transcribed spacers 1 and 2). On the y-axis is an estimate based on the total number of reads mapped to each *de novo* transcriptome assembly.
